# Dynamic profile of SARS-CoV-2 infection among hospitalized patients in Kuwait: a descriptive study

**DOI:** 10.1186/s12879-021-06504-x

**Published:** 2021-08-05

**Authors:** Nada Madi, Ebaa’ Al-Awadhi, Fajer Al-Assaf

**Affiliations:** 1 Virology Unit, Department of Microbiology, Faculty of Medicine, Kuwait University, Kuwait City, Kuwait; 2 Jaber Al-Ahmad Al-Sabah Hospital, Ministry of Health, Kuwait City, Kuwait; 3 Al-Farwaniya Hospital, Ministry of Health, Kuwait City, Kuwait

**Keywords:** SARS-CoV-2, COVID-19, RT-PCR, Kuwait

## Abstract

**Background:**

The coronavirus induced disease 2019 (COVID-19) pandemic caused by the severe acute respiratory syndrome coronavirus 2 (SARS-CoV-2) in Wuhan (China) in December 2019 is currently spreading rapidly worldwide. This study aimed to analyze the dynamic profile of SARS-CoV-2 infection among hospitalized patients that would characterize the period of viral shedding and detection among patients.

**Methods:**

Retrospectively, 103 confirmed SARS-CoV-2 patients hospitalized at Jaber hospital in Kuwait were included. Demographic and clinical characteristics of the patients were collected. Nasopharyngeal swabs were obtained at different time intervals and analyzed by Real-Time RT-PCR for SARS-CoV-2 infection.

**Results:**

Of 103 hospitalized patients with SARS-CoV-2 infection, the median age was 41 years, and 64% were male. The median period from admission to the positive SARS-CoV-2 RT-PCR test was 19 days (IQR, 13–22). The median period from admission to active negative SARS-CoV-2 RT-PCR test result was 22 days (IQR, 16–26). Older patients, patients with comorbidities, and patients with symptoms were more likely to have extended viral shedding.

**Conclusion:**

For the first time, this descriptive study conducted in Kuwait on SARS-CoV-2 RT-PCR test results from 103 patients positive for SARS-provided solid proof and a good understanding of the dynamic profile of SARS-CoV-2 infection among patients in Kuwait. This information will further enrich the global knowledge on the emerging SARS-CoV-2.

## Background

As of 14 March 2021, more than 119 million individuals worldwide were infected with severe acute respiratory syndrome coronavirus type 2 (SARS-CoV-2), the causative agent of coronavirus disease 2019 (COVID-19), and caused approximately 2.6 million deaths (https://coronavirus.jhu.edu/map.html, accessed 14 March 2021). Indeed, SARS-CoV-2 constitutes a significant public health concern due to its high reproduction and transmission rate, lack of immunity in the human population, lack of specific and active treatment and the lack of herd immunity from the recently authorized vaccines [1–3]. Recent reports suggested that even asymptomatic SARS-CoV-2 infected individuals could be the source of the virus [4, 5]. Therefore, rapid and accurate diagnosis, tracing contacts, and isolation of suspected COVID-19 cases are crucial in containing this pandemic [1]. The first confirmed case of SARS-CoV-2 in Kuwait was announced on 24 February 2020 by a group of travellers from Iran. There are 145,204 confirmed cases of SARS-CoV-2, 905 deaths with a 0.6% fatality rate, and 97.1% recovery rate (https://www.coronatracker.com/country/kuwait/, accessed 10-Dec 2020).

SARS-CoV-2 infection is primarily diagnosed based on viral RNA detection by reverse transcriptase-polymerase chain reaction (RT-PCR) in samples from an individual's respiratory tract. So far, there is no descriptive study on the dynamic profile of SARS-CoV-2 in infected patients in Kuwait. Data on SARS-CoV-2 detection at different time points of an infection, including those without any symptoms, will help interpret the RT-PCR test results and to determine the period of viral shedding. Since the SARS-CoV-2 pandemic continues to date, this study's data will help inform Kuwait public health authorities on the right and convenient protocols for quarantine, contact tracing, and isolation. Therefore, this retrospective study aimed to study the dynamic profile of SARS-CoV-2 among infected patients hospitalized in Kuwait and determine the influence of demographic factors on it.

## Methods

### Study population

Retrospectively, 103 hospitalized patients (admission date from 12 March to 11 May 2020) confirmed SARS-CoV-2 infection in Jaber Hospital, Kuwait, were included in this study. Jaber Hospital is one of Kuwait's largest hospitals, assigned to hospitalize all individuals positive with SARS-CoV-2, whether symptomatic or asymptomatic, for centralized isolation following Kuwait policy. All enrolled patients were SARS-CoV-2 RT-PCR confirmed cases, and the patients with signs of COVID-19 had a confirmed diagnosis according to the diagnosis and treatment guidelines for SARS-CoV-2 from the Ministry of Health in Kuwait and the interim guidance from the Centre for Disease Control and Prevention [6]. Test dates and RT-PCR assay results were collected up to the final follow-up date before discharge (3 April to 24 May 2020).

### Data collection

All data, including demographic and clinical information and RT-PCR results for SARS-CoV-2 viral nucleic acid detection, were collected from the electronic medical record system. The collected data were as follow 1. Demographic characteristics, such as age, gender, nationality. 2. Clinical characteristics such as disease outcome (symptomatic or asymptomatic) and comorbidities. 3. SARS-CoV-2 RT-PCR test results. Nasopharyngeal swabs were collected from the patients to detect SARS-CoV-2 viral nucleic acid in sequential time-point during their hospitalization. Positive SARS-CoV-2 RT-PCR assay is defined as the period from the date of the first RT-PCR test to the last positive RT-PCR test result. Active negative SARS-CoV-2 RT-PCR assay is defined as the period from the first RT-PCR test to the first negative RT-PCR test result.

### Real-time reverse transcriptase PCR assays for SARS-CoV-2 RNA detection

Nasopharyngeal swabs were collected from patients suspected of having a COVID-19 infection. According to the manufacturer's protocol, total RNA was extracted using the Roche MagNA Pure LC system (Roche Diagnostics, Indianapolis, IN, USA). The extracted RNA was used for one-step RT-PCR assays of COVID-19 RNA. Two genes, including ORF 1a/b and E genes of SARS-CoV-2, were detected using Cobas® 6800 System and the Cobas® SARS-CoV-2 kit (Roche Diagnostic) according to the manufacturer's protocol. In this technique, the E gene detection was for screening, while ORF1b detection confirmed SARS-CoV-2. Also, LightMix® Modular SARS and Wuhan CoV E-gene and RdRP (RNA-dependent RNA polymerase) gene kits (TIB Molbiol, Berlin, Germany) with LightCycler Multiplex RNA Virus Master mix (Roche, Basel, Switzerland) were used according to the manufacturer's protocol. In this technique, the E gene detection was for screening, while RdRP detection confirmed SARS-CoV-2. Then, RT-PCR was performed on a LightCycler 480 II Real-Time PCR System (Roche Molecular Systems). A cycle threshold value (Ct-value) less than 37 was defined as a positive test result, and a Ct-value of 38 and more was described as a negative test result. Two techniques were used according to the availability of the kits.

### Statistical analysis

All statistical analysis was performed using SPSS version 24.0 (IBM, NY, USA). Medians (interquartile range, IQR) and mean values were calculated for the contentious variables. Categorical variables and percentages were analyzed using the Mann–Whitney *U* test. P values were reported as two-sided, with a significance level of 0.05.

## Results

### Demographic and clinical characteristics of the study population

A total of 103 patients identified as SARS-CoV-2 positive by RT-PCR test were included in this study. The patients, including symptomatic and asymptomatic, were hospitalized in Jaber hospital once they tested positive for SARS-CoV-2 and followed up until discharged. The age of the patients ranged from 1 to 87 years, with a median age of 41 years (IQR, 28–56), compromising 66 (64%) males and 37 (36%) females (Table 1). Among the patients, 54 (52.4%) were Kuwaiti, and 49 (47.6%) were non-Kuwaiti. Asymptomatic individuals represented 49.5%, which is almost half of the study population. According to SARS-CoV-2 [6] guidelines, all symptomatic patients had mild to moderate SARS-CoV-2 infection; however, no patient was transferred to ICU. The most predominant symptoms among patients were fever (75%), followed by cough (55.7%) (Table 1). The sore throat was detected in 21.2% of the patients, headache in 13.5%, shortness of breath in 7.7%, and myalgia in 7.7%. Runny nose was found in 3.8%; however, diarrhoea and vomiting were detected in only 0.9% of the patients. Patients with comorbidities represented 36% of the study population, while the rest (64%) were without comorbidities (Table 1). Among patients with comorbidities, 68% had hypertension, 46% had diabetes mellitus, 16.2% had abstract dyslipidaemia and ischemic heart disease, 13.5% had bronchial asthma, and 8.1% had hypothyroidism, while only 2.7% of the patients had chronic kidney disease and breast cancer (Table 1).

**Table 1:** The demographic and clinical characteristics of the patients (N = 103)

Variable	All patients
Age, median (IQR), y	41 (28–56)
1–17	8 (7.8%)
18–44	50 (48.5%)
45–64	35 (34%)
≥ 65	10 (9.7%)
Gender, n (%)	
Male	66 (64)
Female	37 (36)
Nationality, n (%)	
Kuwaiti	54 (52.4)
Non-Kuwait	49 (47.6)
Asymptomatic, n (%)	51 (49.45
Symptomatic, n (%)	52 (50.5)
Fever	39 (75)
Cough	29 (55.7)
Sore throat	11 (21.2)
Headache	7 (13.5)
Shortness of breath	4 (7.7)
Myalgia	4 (7.7)
Runny nose	2 (3.8)
Diarrhoea	1 (1.9)
Vomiting	1 (1.9)
Without comorbidity, n (%)	66 (64)
With comorbidity, n (%)	37 (36)
Hypertension	25 (68)
Diabetes mellitus	17 (46)
Abstract dyslipidaemia	6 (16.2)
Ischemic heart disease	6 (16.2)
Bronchial asthma	5 (13.5)
Hypothyroidism	3 (8.1)
Chronic kidney disease	1 (2.7)
Breast cancer	1 (2.7)

*IQR* interquartile range

### The dynamic profile of SARS-CoV-2 infection

**Fig. 1:**
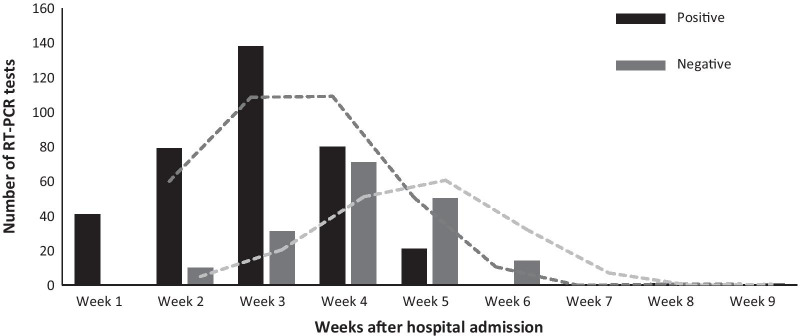
The dynamic profile of SARS-CoV-2 detected by RT-PCR from 103 patients (n = 539 tests). The number of positive and negative results of SARS-CoV-2 RT-PCR tests was summed weeks after the admission

The total number of SARS-RTPCR assay tests performed from 103 patients infected with SARS-CoV-2 was 529 tests, with an average of five tests per patient. The median period from the date of admission to the first SARS-CoV-2 RT-PCR was seven days (IQR, 0–10). The median period from admission to the last SARS-CoV-2 RT-PCR test was 23 days (IQR, 8–28, range, 6–53) (Table 2). The median period from admission to positive SARS-CoV-2 RT-PCR test was 19 days (IQR, 13–22, range, 0–37), while the median period from admission to active negative SARS-CoV-2 RT-PCR test result was 22 days (IQR, 16–26; range, 3–53) (Table 2). The details on the dynamic profile of SARS-CoV-2 infection is shown in Fig. 1A. In the first three weeks after admission, the majority of results of RT-PCR for SARS-CoV-2 were positive, while negative results started to be detected from week two. In week four, the number of positive and negative RT-PCR results were the peak. Nevertheless, from week six onward, both positive and negative RT-PCR results declined.

**Table 2:** SARS-CoV-2 RT-PCR assay results

Variable	Median (IQR), day				
	Total tests (n = 529)	Asymptomatic patients	Symptomatic patients	Patients with comorbidity	Patients without comorbidity
First SARS-CoV-2 RT-PCR	7 (0–10)	7 (0–10)	8 (1–11)	6 (0–10)	7 (1–10)
Last SARS—CoV-2 RT PCR	23 (18–28)	24 (18–28)	22 (17–27)	25 (20–29)	23 (17–26)
Positive SARS-CoV-2 RT-PCR	19 (13–22)	24 (18–28)	22 (17–27)	25 (20–29)	23 (17–26)
Active negative SARS-CoV-2 RT-PCR	22 (16–26)	19 (13–22)	19 (13–22)	23 (17–28)	22 (16–25)

### Influence of age and gender on the dynamic profile of SARS-CoV-2

**Fig. 2:**
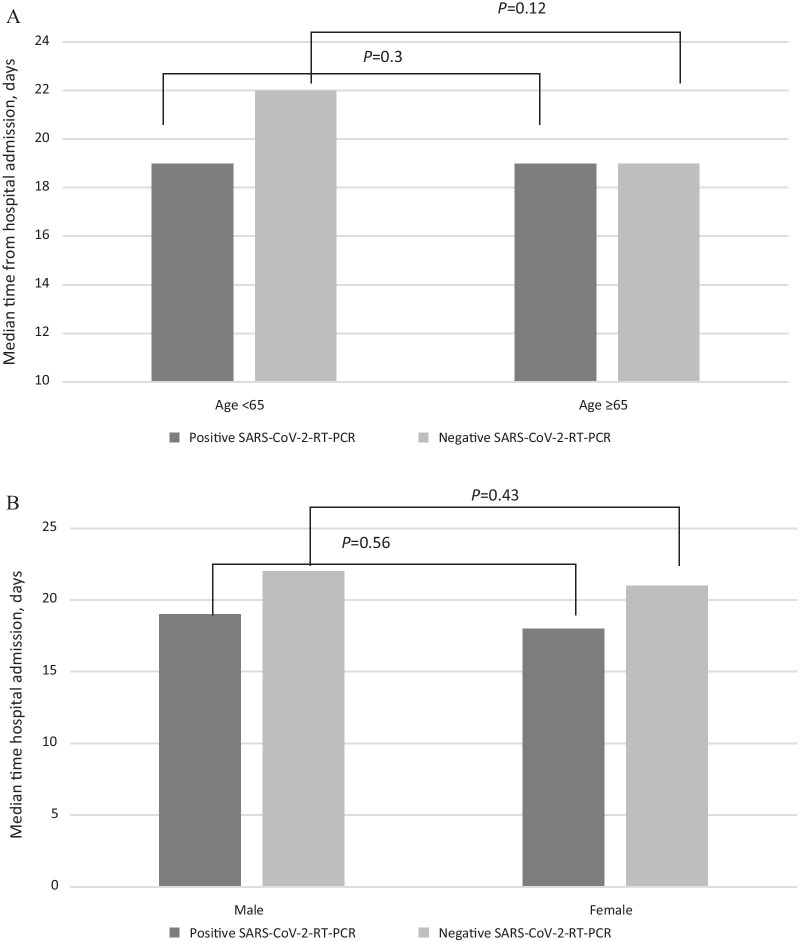
Influence of age and gender on the dynamic profile of SARS-CoV-2. **A** The median period of positive SARS-CoV-2 RT-PCR and active negative SARS-CoV-2 RT-PCR grouped by age. **B** The median period of positive SARS-CoV-2 RT-PCR and active negative SARS-CoV-2 RT-PCR grouped by gender

The impact of age and gender on the dynamic profile of the SARS-CoV-2 RT-PCR assay was also studied. For a more comprehensive analysis, the age group was divided into < 65 and ≥ 65 years. Figure 2A shows no significant difference in the median period of positive SARS-CoV-2 PCR test result between patients aged < 65 and patients aged ≥ 65 years (19 days, IQR, 17–26 and 19 days, IQR, 15–27, *P* = 0.3, respectively). However, the median period of negative SARS-CoV-2 RT-PCR test results in patients aged < 65 years was longer than in patients aged ≥ 65 years, but without significant difference (22 days, IQR, 17–26 and 19 days, IQR, 127, *P* = 0.12, respectively). As shown in Fig. 2B, male patients had a comparable median period of positive and negative SARS-CoV-2 RT-PCR test results to female patients (19 days, IQR, 13–23 vs 18 days, IQR, 13–22, P = 0.56) and (22 days, IQR, 17–26, vs 21 days, IQR, 16–25, *P* = 0.43), respectively.

### SARS-CoV-2 shedding

Based on the period of active negative SARS-CoV-2 RT-PCR test results, the patients were divided into two groups; patients with extended shedding of the virus (≥ 24 days) and patients with non-extended shedding of the virus (≤ 24 days). Only 40 (38.8%) patients had extended viral shedding, while 63 (61.2%) had non-extended viral shedding (*P* = 0.001). As shown in Table 3, patients with extended viral shedding tended to be older. Patients with extended viral shedding were more likely to have comorbidities than patients with non-extended viral shedding, but without a noticeable significant difference (*P* = 0.2). Moreover, patients without comorbidities tended to have non-extended viral shedding, still without a considerable difference (*P* = 0.2). However, the percentage of symptomatic patients who had extended viral shedding was, to some extent, more than the percentage of symptomatic patients who had non-extended viral shedding. Also, asymptomatic patients tended to have non-extended viral shedding, but again without a noticeable difference (*P* = 0.7).

**Table 3:** Viral shedding in SARS-CoV-2 positive patients

Variable	All patients N = 103	Extended shedding (≥ 24 days) n = 40	Non-extended shedding ≤ 24 days) n = 63	P-value
**Age, median (IQR), years**	41 (28–56)	45 (29–56)	40 (27–54)	0.3
**Comorbidity, n (%)**				
Yes	37 (36%)	17 (42.5%)	20 (31.7%)	0.2
No	66 (64%)	23 (57.5%)	43 (68.3%)	0.2
**Symptoms**				
Yes	52 (50.1%)	21 (52.5%)	31 (49.2%)	0.7
No	51 (49.5%)	19 (47.5%)	32 (50.8%)	0.7

I*QR* interquartile range

## Discussion

This is the first descriptive study conducted in Kuwait on SARS-CoV-2 RT-PCR test results from 103 patients positive for SARS-CoV-2. This study provided solid proof and a good understanding of the dynamic profile of SARS-CoV-2 infection among patients in Kuwait during the continuous SARS-CoV-2 pandemic. The first cases of SARS-CoV-2 were recorded in February 2020 from travellers arriving from Iran. At the early phase of the pandemic, all patients detected positive for SARS-CoV-2 by RT-PCR were hospitalized regardless of the infection's outcome (symptomatic or asymptomatic). SARS-CoV-2 infection was monitored during hospitalization by RT-PCR on sequential nasopharyngeal swabs collected from the patients. This study showed that males with a median age of 41 years were the favourable group for the infection. The asymptomatic patients were almost equal in number to the symptomatic patients. It is worth mentioning that the asymptomatic patients in this study never developed any symptoms throughout the hospitalization. Studying asymptomatic individuals' epidemiological significance is essentially better to understand COVID-19 pathogenesis [7, 8]. SARS-CoV-2 generally causes flu-like symptoms, such as fever (93%) [9], non-productive cough (50%) [10], dyspnoea (34.5%), myalgia (27.7%), headache (7.2%), whereas diarrhoea and vomiting may precede these symptoms (6.1%) [9, 10]. Rhinorrhoea was noted in only 4.0% [11] and sore throat in 5.1% [12]. However, the symptoms may become severe and lead to ICU admission, while others die from the complications. All the patients had mild to moderate; 75% developed fever, while 55.7% developed cough, 21.2% had sore throat. Headache was found in 13.5%; however, other symptoms were developed in a lower percentage of the patients, such as shortness of breath (7.7%), myalgia (7.7%), runny nose (3.8%), vomiting and diarrhoea (1.9%). The most prevalent underlying disease among patients positive for SARS-CoV-2 in our study was hypertension (68%), followed by diabetes mellitus (46%). Comorbidities may contribute to the severe and progressive COVID-19[13]. A study by Chen ZM et al*.* (2020) has demonstrated that cardiovascular disease and hypertension were the most common underlying diseases, followed by diabetes mellitus, among adult patients [9].

This study's main goal was to analyze the dynamic profile of SARS-CoV-2 infection among hospitalized patients that would characterize the period of viral shedding and detection among patients. Our results showed that the median period between hospital admission and positive SARS-CoV-2 RT-PCR test result was 19 days (IQR, 13–22). However, there were no significant differences in the period of viral detection between patients with symptoms and patients without symptoms, suggesting the possibility of transmission by asymptomatic patients. Also, the significant differences in viral detection period between patients with comorbidities and patients without comorbidities were lacking. The median period for active negative (first negative) RT-PCR test results was 22 days (IQR, 16–26), without noticeable differences between different patients' categories. We showed that most RT-PCR tests were positive at week three with a gradual decrease trend after that (Fig. 1). On the other hand, the negative results of RT-PCR tests start to increase from week two after admission, peaked at week four and then began to decrease until the end of follow-up (6 weeks) (Fig. 1). In parallel to our results, a study reported a median period of viral shedding of 20 days (range, 8–37 d) in191 patients with COVID-19 [14]. However, a study in Singapore in patients infected with SARS-CoV-2 showed that the period of viral shedding was extended to 24 days after symptom onset [15]. The studies on the duration of virus shedding in symptomatic and asymptomatic patients were varied [16, 17]. In parallel to our results, Zhuo et al*.* have shown that the duration of viral shedding remained similar in the symptomatic and asymptomatic patients (7 days) [18].

However, Yongchen et al. have shown that the median period of virus detection in asymptomatic patients was longer (18 days) compared with patients with severe disease (14 days) and patients with mild symptoms (10 days) [19]. Other studies showed that the period of SARS-CoV-2 detection in hospitalized asymptomatic patients ranged from 7 to 23 days [17, 20].

We studied the influence of demographic factors on the dynamic profile of SARS-CoV-2 infection. Our results showed no significant differences in the median duration of positive or negative SARS-CoV-2 results between patients aged < 65 and ≥ 65 or between males and females. In contrast to our study, a study has shown that infected older patients (≥ 65 years old) had a significantly extended period of positive SARS-CoV-2 RT-PCR test results than patients < 65 years [21]. Another study has shown that the median SARS-CoV-2 viral positivity duration was significantly shorter in younger patients [22].

The correlation between clinical characteristics and the duration of viral shedding in patients positive for SARS-CoV-2 was also explored. Our results showed that patients with extended viral shedding (≥ 24 days) are likely to be older and have comorbidities. Also, patients with symptoms had a slightly more extended period of viral shedding than patients without symptoms. Studies on the association between the duration of viral shedding and older age showed inconsistent results, where some studies showed positive associations [21, 23, 24], while others showed no association [14].

Our study has several limitations. At the beginning of the pandemic, the accuracy of SARS-CoV-2 varied; however, significant improvements in the detection protocol and sampling experience were achieved until the current time. Second, information on the viral load, which is lacking in this study, will reflect the virus's infectivity during the disease course. Third, this study only investigated the profile of SARS-CoV2 infection in nasopharyngeal swabs, while other samples such as different respiratory samples, blood and stools samples should be included. Finally, this study's lack of virus culture data reduced its credibility in viral infectivity during the disease course. RT-PCR is considered the gold standard technique for detecting SARS-CoV-2 due to its high sensitivity and specificity rates and the ability to quantify the virus. However, the technique has some limitations—first, the possibility of false-positive results may range from 2 to 37%. Second, many diagnostic platforms that target spike (S) gene showed false-negative results due to the emergence of new variants of SARS-CoV-2 such as B.1.1.7 lineage. Another diagnostic tool that has a crucial role in diagnosing COVID-19 is chest computed tomography (CT). CT has a role in detecting pulmonary changes among COVID-19, monitoring disease progression, and guiding therapy [25].

## Conclusion

In summary, this is the first study in Kuwait that demonstrated the dynamic profile of SARS-CoV-2 infection by analyzing the test results of the SARS-CoV-2 RT-PCR assay. The median duration of viral detection of 19 days after onset of symptoms and hospital admission, and older age and comorbidities may have more extended viral shedding and more complications. This information will further enrich the global knowledge on the emerging SARS-CoV-2. We recommend a more extensive scale study involving many patients to understand the dynamic profile of SARS-CoV-2 infection better.

## Data Availability

The datasets used and/or analyzed during the current study are available from the corresponding author on reasonable request.

## Abbreviations

COVID-19: Coronavirus induced disease 2019
SARS-CoV-2: Severe acute respiratory syndrome coronavirus 2
RT-PCR: Reverse transcriptase-polymerase chain reaction
